# The association of menopause with cardiometabolic disease risk factors in low- and middle-income countries: a systematic review and meta-analysis

**DOI:** 10.1097/GME.0000000000002292

**Published:** 2023-12-18

**Authors:** Raylton P Chikwati, Tinashe Chikowore, Nasrin Goolam Mahyoodeen, Nicole G Jaff, Jaya A George, Nigel J Crowther

**Affiliations:** 1 Department of Chemical Pathology, Faculty of Health Sciences, University of the Witwatersrand, Johannesburg, South Africa; 2 Sydney Brenner Institute for Molecular Bioscience, Faculty of Health Sciences, University of the Witwatersrand, Johannesburg, South Africa; 3 SAMRC/Wits Developmental Pathways for Health Research Unit (DPHRU), Faculty of Health Sciences, University of the Witwatersrand, Johannesburg, South Africa; 4 Department of Internal Medicine, Chris Hani Baragwanath Academic Hospital, Faculty of Health Sciences, University of the Witwatersrand, Johannesburg, South Africa; 5 Department of Chemical Pathology, National Health Laboratory Service, Johannesburg, South Africa; 6 The Diagnostic Innovation Hub (DIH), University of the Witwatersrand, Johannesburg, South Africa; 7 Channing Division of Network Medicine, Brigham and Women’s Hospital, Boston, MA, United States of America; 8 Harvard Medical School, Boston, MA, United States of America

**Keywords:** Premenopause, postmenopause, cardiometabolic disease risk factors, low- and middle-income countries.

## Abstract

**Importance:**

Menopause is an integral part of women’s health and studies in high income countries have shown an increase in CMD risk factors in post- compared to premenopausal women. However, to date no study has combined and assessed such studies across LMICs. This would better inform early monitoring and intervention strategies for reducing CMD risk factor levels in midlife women in these regions.

**Objective:**

To evaluate evidence from the literature on differences in CMD risk factors between pre- and post menopausal midlife women living in LMICs.

**Evidence Review:**

A systematic review with meta-analysis of original articles of all study designs from the databases PubMed, PubMed Central, Scopus, and ISI Web of Science was conducted from conception until April 24, 2023. Studies that met the inclusion criteria were included in the analysis. Quality assessment of the articles was done using the Newcastle-Ottawa Scale, adapted for each study design. The study protocol was registered with the International Prospective Register of Systematic Reviews and adheres to the Preferred Reporting Items for Systematic Reviews and Meta-Analysis. For the meta-analysis, fixed-effects models were used to pool the odds ratios (ORs), as measures of association.

**Findings:**

Our search identified 4,849 relevant articles; 44 for the systematic review and 16 for the meta-analysis, in accordance with our inclusion criteria. Compared with premenopausal women, the postmenopausal stage was associated with metabolic syndrome (OR=1.18 (95 % CI 1.11–1.27)), high waist-to-hip ratio (OR=1.22 (95% CI 1.12–1.32)), hypertension (OR=1.10 (95% 1.04–1.16)), elevated triglycerides (OR=1.16 (95% CI 1.11–1.21)) and elevated plasma glucose (OR=1.21 (95% CI 1.15–1.28)).

**Conclusions and Relevance:**

This study confirmed that CMD risk factors are present at higher levels in post- than premenopausal women. This demonstrates an urgent need for public health policies that focus on early monitoring and interventions targeted at reducing CMD risk and related adverse outcomes in midlife women in these nations.

## 1 Introduction

Studies have shown that hormonal changes along the hypothalamus-pituitary-ovarian axis during the menopause transition (MT) may be associated with adverse changes in cardiometabolic health in midlife women.^1,2^ One report from the Study of Women Across the Nation (SWAN), highlighted that despite the levels of total testosterone (T) remaining constant during the MT, the more rapid decline of estradiol (E2) creates a more androgenic sex hormone profile termed the relative androgen excess, which contributes to increased risk of the metabolic syndrome.^3^ Reports have also shown that declining E2 and increasing follicle stimulating hormone (FSH) levels during the MT are associated with drastic changes in body fat composition and distribution.^4,5^ These changes have been associated with central obesity and increased secretion of pro-inflammatory adipokines and free fatty acids which in turn increase the risk of insulin resistance, and hypertension.^6,7^

Studies have shown a higher prevalence of obesity among women compared with men from LMICs, and these differences are reported to be more apparent in midlife than in childhood years.^8^ As a result, an in-depth analysis of the contribution of menopause to obesity and associated CMD risk factors in women in LMICs is warranted. Furthermore, a meta-analysis showed that women from LMICs reach menopause at an earlier age than those from high-income countries (HICs).^9^ In this meta-analysis involving thirty-six studies across the six continents, the mean (with 95% CIs) age at menopause was lower in Africa (48.4 (48.1–48.7)), Latin America (47.2 (45.9–48.6)), Asia (48.8 (48.1– 49.4)), and the Middle East (47.4 (46.9–47.8)), compared to Australia (51.3 (49.8–52.8)), Europe (50.5 (50.0–51.1)) and the United States (49.1 (48.8–49.4)).^9^ Early age at menopause has been linked with increased CMD risk factors,^10^ therefore suggesting heightened risk in LMICs.

At present, there are no data quantifying the differences between the levels of CMD risk factors in pre- and postmenopausal women in studies from LMICs despite an increasing prevalence of obesity and associated CMD in these countries. The objective of this systematic review and meta-analysis was therefore to evaluate evidence from the literature on the links between menopause and CMD risk factors in midlife women living in LMICs.

## 2 Methods

### 2.1 Protocol

This systematic review and meta-analysis were performed using the Preferred Reporting Items for Systematic Review (PRISMA) 2020 guidelines and was registered with the International Prospective Register of Systematic Reviews (PROSPERO) with the number CRD42021295401.^11^

### 2.2 Search strategy and data sources

We searched the databases; PubMed, PubMed Central, Scopus, and ISI Web of Science, for original articles of all study designs from inception until April 24, 2023. The query terms consisted of the key words related to “premenopause”, “postmenopause”, “cardiometabolic disease risk factors” and “LMICs”. The search strategy is fully detailed in the Supplemental Table 1.

### 2.3 Eligibility criteria

We only included studies conducted in LMICs as defined by the World Bank list of economies (June 2020).^12^ These studies assessed differences in CMD risk factors between pre- and postmenopausal women. The inclusion criteria were: 1) studies that enrolled both pre- and postmenopausal women, 2) studies evaluating differences in CMD risk factors according to the menopausal stage, and 3) studies published in English. Articles were excluded if they were reviews, editorials, or preliminary reports.

### 2.4 Data extraction

**Figure 1:**
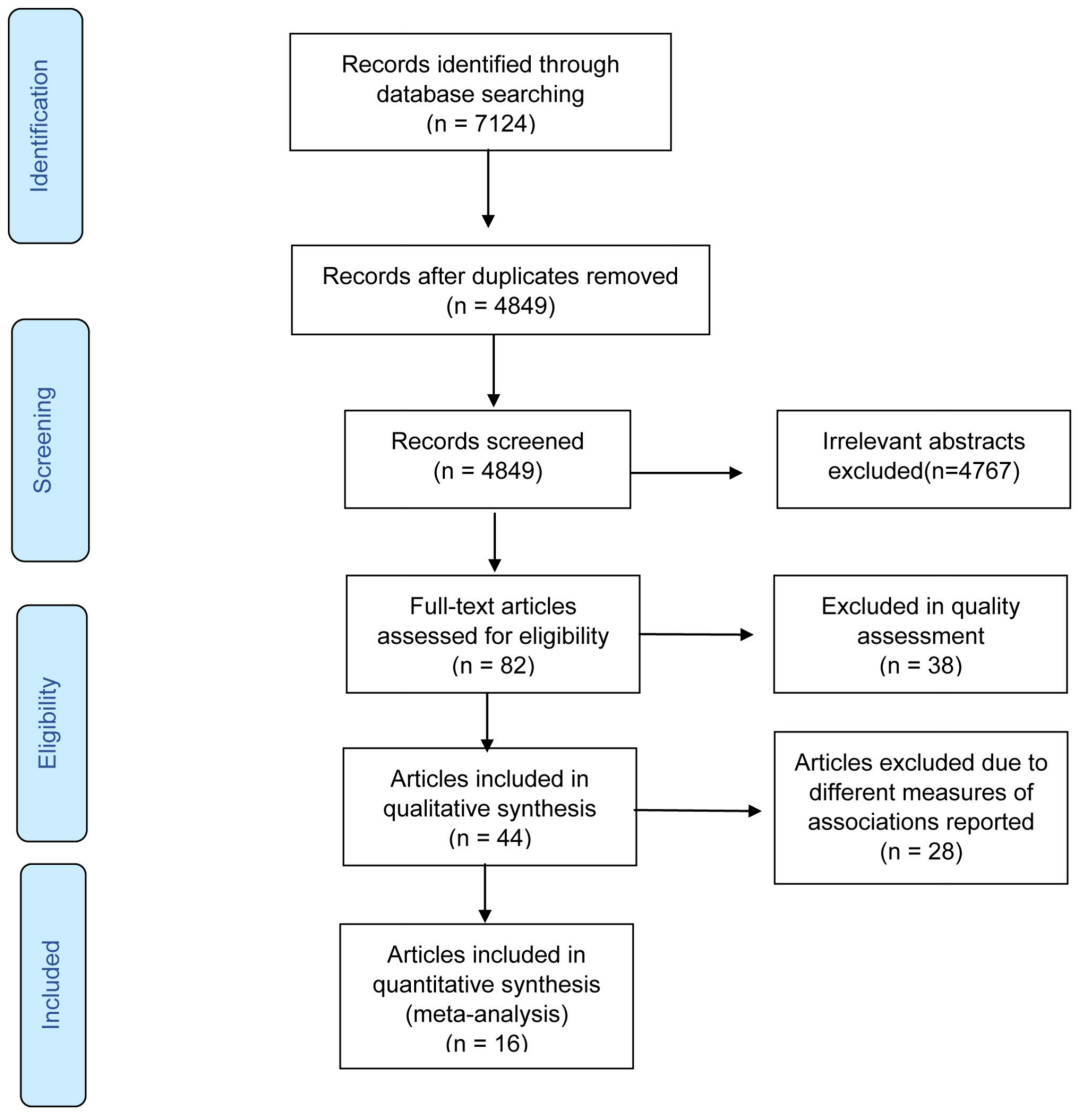
PRISMA flow chart of literature screening and selection. PRISMA - preferred reporting items for systematic reviews and meta-analysis

One researcher (RPC) independently screened all initially identified articles and abstracts using the Rayyan software.^13^ The number of included and excluded records is mapped in Figure 1. Studies deemed to potentially meet inclusion criteria underwent a full-text assessment by two independent reviewers (RPC and NGM). The consensus between two authors satisfied the inclusion criteria. Disagreements were resolved by a third reviewer, NJC.

#### 2.2.4 Quality Assessment

Two reviewers, RPC and NGM, independently used the modified Newcastle-Ottawa Scale (NOS)^14^ to assess the methodological quality of selected articles. Two separate NOS tools developed for cross-sectional and longitudinal studies were used in the quality assessment. Based on the total score, the risk of bias was assigned into two categories: low risk (7–9) and high risk (0–6). Only studies with a low risk of bias were included in this study. Any disagreements were referred to a third reviewer, NJC.

### 2.5 Statistical Analyses

To quantitatively assess the association between menopause stage and CMD risk factors i.e. metabolic syndrome (MetS), blood pressure, triglycerides, HDL-C, blood glucose, and carotid intima thickness (cIMT) levels, obesity, waist circumference (WC), waist-to-hip ratio (WHR) and type 2 diabetes mellitus, we calculated the pooled estimates of odds ratios and associated 95% confidence intervals using the inverse variance fixed-effect model. In the analyses, studies were grouped based on the defined outcome of interest (CMD risk factor).

Heterogeneity between studies was assessed using Cochran’s Q statistic (p<0.01 indicative of heterogeneity) and the I^2^ index (values 25%, 50% and 75% suggestive of low, moderate, and high heterogeneity, respectively). All statistical analyses were performed using Stata 16.1 (StataCorp LLC, College Station, TX).

## 3 Results

### 3.1 Search results

Figure 1 shows the PRISMA flow chart on the screening and selection of the research articles. Briefly, the initial search identified 7,124 abstracts. After removing duplicates, 4,849 titles and abstracts were screened. Of these, 4,767 irrelevant articles were excluded, leaving 82 articles for full-text review. Thirty-eight of the 82 articles were excluded in the quality assessment. As a result, 44 articles constituted the systematic review. Of these 44 articles, 16 were eligible for the quantitative analysis and 28 were excluded due to the following reasons: reporting of a CMD risk factor that was uncommon to other articles (n=3), no combined comparison of pre- and postmenopausal stages on CMD risk factors (n=1), different definition criterion for MetS (n=1), and studies that did not report odds ratios as measures of association (n=23).

### 3.2 Study characteristics and populations

Tables 1 and 2 show the characteristics of the 44 studies included in the systematic review. The studies were from the following countries: China^15–27^ (n=14), Brazil^28–34^ (n=7), Iran^35–43^ (n=9), India^44–46^ (n=3), Tunisia^47–49^ (n=3), Thailand^50,51^ (n=2), Mexico ^52^ (n=1), the Democratic Republic of Congo^53^ (n=1), Ghana^54^ (n=1), South Africa^55^ (n=1), Bangladesh^56^ (n=1), and Chile^57^ (n=1). In total, the studies comprised of 353,589 participants, with sample sizes ranging from 122 to 281,319. Staging of natural menopause in all the studies was performed by asking the study participants about their menstrual history, with slight variations in 3 articles^31,51,52^ where additional confirmation was done by measuring the levels of the sex hormones oestradiol and follicle stimulating hormone. In 31 articles, the differences between CMD risk factors were compared between two menopausal stages namely, the pre- and postmenopause. In these studies, premenopause was defined as regular menses while postmenopause was amenorrhea for 12 consecutive months. In the remaining 13 articles, a third menopausal group, the perimenopause group was included. Perimenopause was defined as irregular menses within the past 12 months. Women who had a history of surgical menopause were excluded from most of the reviewed articles. Only 4 articles^15,16,24,56^ in this review included participants with a known history of surgical menopause. Furthermore, the use of hormone therapy (HT) was confirmed in only 4 articles^30,31,40,43^. Articles were later grouped according to each CMD risk factor as shown in Table 1.

Table 2 presents the 44 articles included in the systematic review. In the 17 articles describing the MetS, 11 showed higher MetS in post- than premenopause^15,19,20,27,34,40,41,45,49,56^, and 6 showed no differences^19,28,31,32,39,50^. In the 14 articles focused on obesity, 3 showed higher obesity risk in post- than premenopause,^29,35,46^ and 11 showed no differences^17,22,24,29,30,37,48,55,57,58^. One of these studies^55^ also measured total body fat mass which was higher in post- than premenopausal women. In the 11 articles on WC and WHR, 5 showed that postmenopausal women had higher WC^16,24,30,47,58^ and 4 articles showed higher WHR^21,22,30,58^, but no menopausal differences were reported on WC in 4 studies^29,37,42,55^. One of these studies showed no difference in abdominal subcutaneous and visceral fat between the menopause groups^55^. In the 12 articles on blood lipids, HDL-C was lower in post- than in premenopause in one study^36^, but no differences were reported in triglycerides in a separate study^42^. Elevated total cholesterol, LDL-C, lipoperoxides, and triglyceride-rich lipoprotein-cholesterol (TLR-C) levels were reported in 9 articles^16,21–23,33,38,43,44,52^ in post- compared to premenopausal women. In the 10 articles on blood glucose and insulin levels, 3 showed higher glucose levels in post- than premenopausal women^44,47,48^ but 5 showed no difference,^16,21,22,24,37^ 2 showed higher insulin in post- than premenopausal women^47,48^ and in 2 studies diabetes was more prevalent in postmenopausal women^21,25^. In the 9 articles on blood pressure, 6 showed higher blood pressure levels in post- than premenopause^16,23,24,44,46,47^ and 3 showed no differences.^21,22,37^ In the 2 articles describing cIMT, postmenopausal women had higher cIMT levels than their premenopausal counterparts^18,51^ and in 1 study 10-year risk of cardiovascular disease was higher postmenopausally.^26^

In the meta-analysis, 16 studies from the following countries, China^15–18,21,27^ (n=7), Brazil^28,30,32^ (n=3), Tunisia^47–49^ (n=3), Thailand^50,51^ (n=2), Bangladesh^56^ (n=1), and Iran (n=1)^37^ constituted a total of 29,361 women. Studies were further categorised according to standard definitions of the CMD risk factors as follows: 1) MetS defined by the National Cholesterol Education Program Expert Panel on the Detection, Evaluation, and Treatment of High Blood Cholesterol in Adult Treatment Panel III (NCEP-ATP III criteria)^28,32,48–50,56^ (n=6), 2) elevated serum triglycerides (≥1.69 mmol/L)^15,16,49,56^ (n=4), 3) elevated fasting glucose (≥6.1 mmol/L)^15,49,56^, 4) low HDL-C (<1.29 mmol/L)^15,56^ (n=2), 5) hypertension (SBP ≥140 mmHg, DBP ≥90 mmHg and use of antihypertensives)^16,21,27,37,47^ (n=5), 6) hypertension (SBP ≥135 mmHg, DBP ≥85 mmHg and/or use of antihypertensives)^15,49,56^ (n=3), 7) high WC (≥80 cm)^15,16^ (n=2) and 8) high WC (≥88 cm)^30,49,56^ (n=3), 9) high WHR (≥0.86)^17,30^ (n=2), and 10) obesity (BMI ≥28kg/m^2^)^16,17^ (n=2).

### 3.3 Primary outcomes

**Figure 2:**
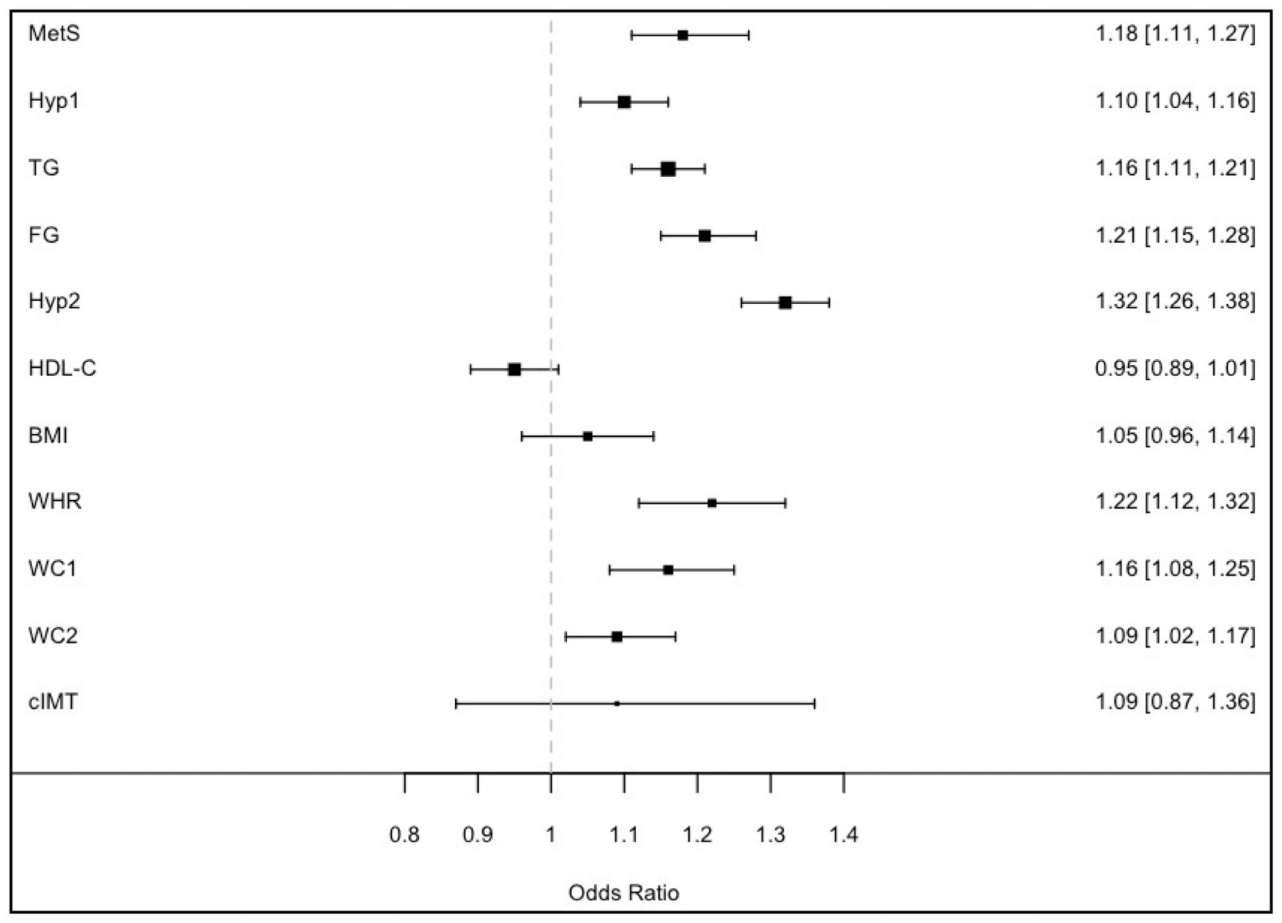
Meta-analysis of studies showing differences in cardiometabolic disease risk factors according to menopausal stage High BMI (body mass index) (≥28 kg/m^2^), elevated cIMT (carotid intima media thickness) (≥0.70mm), elevated FG (fasting glucose) (≥6.1 mmol/L), low HDL-C (high density lipoprotein cholesterol) (<1.29 mmol/L), Hyp (hypertension) 1 (systolic BP ≥140 mmHg and diastolic BP ≥90 mmHg), Hyp 2 (systolic BP ≥130 mmHg and diastolic BP ≥85 mmHg), MetS (metabolic syndrome) (NCEP ATP III definition), high TG (triglycerides) (≥1.69 mmol/L), high WC (waist circumference) 1 (≥80 cm), high WC2 (≥88 cm), and high WHR (waist-to-hip ratio) (≥0.85). Odds ratios presented as post- vs. premenopausal stage.

Figure 2 shows the combined effect size estimates in studies that evaluated differences in CMD risk factors according to menopausal stage. Overall, postmenopausal stage was associated with greater CMD risk as supported by significant odds ratios for MetS, hypertension and high triglyceride, fasting blood glucose, waist circumference, and WHR levels. However, odds ratios were not significant for BMI, HDL-C and cIMT levels in post- relative to premenopausal stage (Figure 2). The individual forest plots for each CMD risk factor are shown in the Supplemental Figures 1–11.

#### 3.3.1 Metabolic Syndrome

Six studies involving 5,177 women from Brazil^28,32^ (n=2), Thailand^50^ (n=1), Tunisia^48,49^ (n=2), and Bangladesh^56^ (n=1) were included in the meta-analysis for MetS. Pooled analysis of these studies showed that the risk of MetS was higher in post- than premenopausal women (OR=1.18; 95% CI, 1.11–1.27, P=0.19, and I^2^=33.4% (Figure 2 and Supplemental Figure 1; with a moderate heterogeneity between the studies).

#### 3.3.2 Blood Pressure

Five studies involving 16,602 women from China^16,21,27^ (n=3), Tunisia^47^ (n=1), and Iran^37^ (n=1) showed that when using a definition of hypertension of SBP ≥140 mmHg and/or DBP ≥90 mmHg, postmenopausal women had a higher risk of hypertension compared to their premenopausal peers (OR=1.10; 95% CI 1.04–1.16, P=0.22, and I^2^ =29.9%) (Figure 2 and Supplemental Figure 2, with a moderate heterogeneity between the studies). A similar trend was shown in 3 studies ^15,49,56^ that defined hypertension as SBP ≥130 mmHg and DBP ≥85 mmHg (OR=1.32; 95% CI 1.26–1.38, P<0.001, and I^2^ =97.9%) (Figure 2 and Supplemental Figure 3), however these studies showed a highly significant level of heterogeneity.

#### 3.3.3 Triglycerides and HDL-C

Four studies involving 13,465 women from China^15,16^ (n=2), Bangladesh^56^ (n=1), and Tunisia^49^ (n=1) showed that the risk of elevated triglyceride levels (≥1.69 mmol/L) was higher in post-than in premenopausal women (OR=1.16; 95% CI 1.11–1.21, P<0.001, and I^2^ =87.7%) (Figure 2 and Supplemental Figure 4). When low HDL-C levels (<1.29 mmol/L) were compared in two studies ^15,56^ with a combined sample size of 4,313 women, no differences were present between pre- and postmenopausal women (OR=0.95; 95% CI 0.89–1.01, P=0.001, and I^2^ =91.6%) (Figure 2 and Supplemental Figure 5). All these analyses showed a high level of heterogeneity.

#### 3.3.4 Glucose

Three studies involving 5,274 women from China^15^, Bangladesh^56^ and Tunisia^49^ showed that the odds ratio of impaired blood glucose levels (≥6.1 mmol/L) was higher in post- than premenopausal women (OR=1.21; 95% CI 1.15–1.28, P=0.001, and I^2^=91.1%) (Figure 2 and Supplemental Figure 6), but with a high level of heterogeneity.

#### 3.3.5 Obesity

Pooled results of two studies from China^16,17^ involving 13,654 women showed that the risk of obesity (BMI ≥28 kg/m^2^) was similar in pre- and postmenopausal women (OR=1.05; 95% CI 0.96–1.14, P=0.13, and I^2^ =56.8%) (Figure 2 and Supplemental Figure 7), with a moderate level of heterogeneity between the studies.

#### 3.3.6 Waist circumference and waist-to-hip ratio

In two studies from China^15,16^ involving 10,702 women, postmenopausal women had an increased WC (≥80 cm) than their premenopausal peers (OR=1.16; 95% CI 1.08–1.25, P=0.02,and I^2^=81.9%) (Figure 2 and Supplemental Figure 8). A similar trend was observed when three studies from Brazil^30^, Bangladesh^56^, and Tunisia^49^, that defined high WC as ≥88 cm were meta-analysed (OR=1.09; 95% CI 1.02–1.17, P=0.01, and I^2^=77.3%) (Figure 2 and Supplemental Figure 9).Furthermore, pooled analyses from two studies from China^17^ and Brazil^30^ showed that postmenopausal women had higher WHR (≥0.85) than premenopausal women (OR=1.22; 95% CI 1.12–1.32, P=0.14, and I^2^ =54.5%) (Figure 2 and Supplemental Figure 10). The level of heterogeneity between all these studies was moderate to high.

#### 3.3.7 cIMT

In two studies from China^18^ and Thailand^51^ involving 2,253 women, there were no differences in the risk of high cIMT levels (≥0.70mm) between post- and premenopausal women (OR=1.09; 95% CI 0.87–1.36 *P*=0.09, and I^2^=64.4%) (Figure 2 and Supplemental Figure 11). There was a moderate level of heterogeneity across these studies.

## 4 Discussion

This systematic review and meta-analysis on midlife women from LMICs show that the postmenopausal stage is associated with higher risk of MetS, elevated triglycerides, elevated blood glucose, high blood pressure, and high waist circumference but no differences when obesity, HDL-C and cIMT levels were compared between the two menopausal groups. These observations highlight a disproportionate burden of CMD risk factors in post- compared to premenopausal women in LMICs.

Our study broadens the understanding of the association of menopause with CMD risk factors by combining studies from LMICs into a large sample size (40 517 participants). Our findings are similar to a meta-analysis on MetS which included studies from around the world ^59^. In their analysis, postmenopausal women were 3.5 times more likely to develop MetS compared to premenopausal women.^59^ Furthermore, the higher prevalence of the individual components of MetS in post- than in premenopausal women observed in that study, corroborate our findings.

In longitudinal studies from HICs, menopause has been shown to have differential effects on CMD risk factors. In the SWAN study, MetS, total cholesterol, LDL-C, HDL-C, and apo-B lipoproteins were independently associated with menopause only in the first year after FMP.^1,2^ The study also showed no influence of menopause on BMI, blood glucose, insulin, triglyceride, and blood pressure levels.^2^ In the Atherosclerosis Risk in Communities (ARIC) study, the progression of MetS was rapid during the MT but it decreased after the FMP, which was more prominent in African Americans than White women.^60^ In the Melbourne Women’s Midlife Health Project (MWMHP) study, HDL-C levels increased around the first year before FMP but decreased in the first year postmenopause.^61^ Other changes in blood lipids (triglycerides and LDL-C), BMI and diastolic blood pressure were only related to chronological ageing or one of the traditional risk factors.^61^ Furthermore, the Radiation Effects Research Foundation (RERF) study showed that total serum cholesterol levels increased from three years before FMP to one-year post-FMP whereas increased BMI and systolic blood pressure were associated with chronological ageing but not menopause.^62^ Guthrie *et al*., observed that women gained an average of approximately 2.1 kilograms over five years, but these differences were not menopause related. However, the study showed that waist circumference and waist-hip ratio increased with MT.^63^ There are many possible reasons for these different outcomes across studies, as also observed in the current systematic review, including differences in sample size, ethnicity, and time points at which CMD risk factors were measured. However, it is interesting to note that in these studies changes in BMI were not related to the menopause but changes in waist and WHR were, and this was also observed in the current meta-analysis.

The differences in CMD risk factor levels between pre- and postmenopausal women may relate to hormonal changes during the MT. In the SWAN study, menopause was associated with increasing bioavailable T, and declining E2 and sex hormone binding globulin (SHBG) levels.^1^ The changes in testosterone and SHBG were associated with the MetS and its components. However, neither baseline E2 levels nor its decline during menopausal transition was associated with MetS.^1,60^ In the age-adjusted analyses, the T:E2 ratio and free androgen index (FAI) increased by approximately 10% from baseline over the five years of follow-up. Supporting evidence from one meta-analysis study showed that women with type 2 diabetes mellitus had higher T but lower SHBG levels than controls ^64^. It is hypothesised that the association between SHBG and MetS is mediated by the inhibitory effect of insulin on the synthesis of SHBG.^65^ The association of sex hormone levels with CMD risk factors during menopause indicates that hormone therapy may be a useful intervention strategy for these diseases. However, the feasibility of using hormone therapy is debatable in under-resourced healthcare systems and very few studies have investigated its use in such environments. In a large cross-sectional study across 11 Latin American countries, the Collaborative Group for Research of the Climacteric in Latin America (REDLINC) showed that the current use of menopausal hormonal therapy (MHT) was associated with reduced risk of MetS.^66^ Furthermore, a study from Brazil showed that the use of MHT was associated with a lower risk for hypertension.^67^ However, these were cross-sectional studies and the use of MHT in these studies was low (12.5%).^66^

### 4.1 Limitations and strengths

The present study has some limitations. Firstly, the number of identified articles per CMD risk factor in our analyses were small; thus, we could not investigate sources of heterogeneity further. Secondly, the studies assessed in the meta-analysis were dominated by large studies from China with none available from sub-Saharan Africa. Thirdly, our analyses were based on observational data and were therefore limited by study design as far as potential unmeasured confounders and direction of associations were concerned. Despite this, our study provides a comprehensive review of the current literature on this topic in LMICs and was guided by a registered protocol.

### 4.2 Conclusions

The results of this systematic review and meta-analysis show that menopause is associated with an increased risk for CMD risk factor levels in LMICs. Therefore, it is important to focus on prevention strategies such as lifestyle and behavioural changes to mitigate the development of CMD in midlife women in these countries. However, it must be noted that this analysis included a small number of studies with high levels of heterogeneity. More studies are therefore required in LMICs to investigate the relationship of menopause with CMD risk factors and to develop cost-effective interventions for these diseases.

## Supplementary Material

Supplemental Data File (.doc, .tif, pdf, etc.)

Supplemental Video File

## Figures and Tables

**Table 1:** CMD risk factors and corresponding articles examined

CMD risk factor	Number of articles examined
MetS	17
Obesity	14
Blood lipids	12
WC and WHR	11
Blood glucose and insulin levels	11
BP	9
cIMT	2
Others	4

BP-blood pressure, cIMT-carotid intima media thickness, CMD-cardiometabolic disease, DM-diabetes mellitus, MetS-metabolic syndrome, Others-Fat mass, visceral and subcutaneous adipose tissues, WC-waist circumference, and WHR-waist hip ratio.

**Table 2:** Studies included in the qualitative analyses

First Author, Year, Reference	Country	Study Type	Age, y	Sample Size	Outcome	Main Results
Neto (2010)^28^^a^	Brazil	Cross-sectional	40–65	323	MetS	Influence of age on MetS was prevalent, and attenuated any menopausal differences
Moreira (2020)^32^^a^	Brazil	Cross-sectional	45–74	419	MetS	No association between menopausal stage and MetS
Jesmin (2013)^56^^a^	Bangladesh	Cross-sectional	40.0±14.0	1802	MetS	MetS higher in post vs premenopausal women
Belfki (2012)^49^^a^	Tunisia	Cross-sectional	50.3±9.6	961	MetS	Postmenopausal stage was associated with higher risk of MetS
Jeenduang (2014)^50^^a^	Thailand	Cross-sectional	48.8±11.0	361	MetS	No association between menopausal stage and MetS
Ali (2014)^48^^a^	Tunisia	Cross-sectional	49.5±9.6	1311	BP, obesity, glucose, and insulin resistance	Only hyperglycemia was associated with postmenopausal stage
Ren (2019)^16^^a^	China	Cross-sectional	56 (47–65)	8191	BMI, TGs, glucose, BP, WC	Menopause associated with increased risk of higher BMI, hypertension, TGs, and WC
He (2012)^21^^a^	China	Cross-sectional	50.1±5.4	4743	BMI, WHR, lipids, glucose, BP	Elevated total cholesterol, LDL-C, triglycerides, and waist hip ratio were the only risk factors associated with postmenopausal status.
Zhou (2014)^27^^a^	China	Cross-sectional	53.4±10.3	6324	MetS	Postmenopausal status was a risk factor for hypertension
Ali (2016)^47^^a^	Tunisia	Cross-sectional	56.1±9.4	242	BMI, WC, BP, glucose, HOMA, lipids	Waist circumference, HOMA, and apo B levels were associated with hypertension in postmenopausal women.
Tehrani (2013)^37^^a^	Iran	Longitudinal	Baseline: 38.6±4.6	675	BMI, glucose, lipids, WC, BP	Only LDL-C and total cholesterol were associated with postmenopause
Zhou (2018)^15^^a^	China	Cross-sectional	53.3±10.3	6022	MetS	MetS was higher in postmenopausal women
Chen (2020)^17^^a^	China	Cross-sectional	44.7±12.9	5373	Obesity	Menopause was a risk factor for central and visceral obesity but not general obesity
Donato (2006)^30^^a^	Brazil	Cross-sectional	40–55	358	WC, WHR, BMI	Postmenopausal women had higher WC & WHR than premenopausal women
Ieamtairat (2019)^51^^a^	Thailand	Cross-sectional	49.3±2.0	122	cIMT	Menopause was associated with increased cIMT levels
Zhou (2015)^18^^a^	China	Cross-Sectional	40–65	2131	cIMT	Postmenopausal had higher cIMT levels than premenopausal women
Montazeri (2018)^35^	Iran	Longitudinal	Baseline: 43±5	929	BMI	Menopause was associated with increasing BMI.
Nazari (2003)^36^	Iran	Longitudinal	Baseline: 30–74	3778	HDL-C	HDL-C associated with coronary heart disease in postmenopause
Tehrani (2014)^38^	Iran	Longitudinal	20–50	755	Lipids	Dyslipidemia associated with lower AMH levels
Heidari (2010)^39^	Iran	Cross-sectional	45–70	1596	MetS	Menopause was only associated with elevated triglycerides.
Maharlouei (2014)^40^	Iran	Cross-sectional	52.2±8.4	924	MetS	Menopause was associated with higher prevalence of MetS
Ainy (2007)^41^	Iran	Cross-sectional	45–65	2182	MetS	Menopause was associated with higher prevalence of MetS
Sarrafzadega (2013)^42^	Iran	Cross-sectional	30–60	4146	TGs, WC	Menopause was not associated with a high triglyceride/waist circumference phenotype
Yousefzadeh (2013)^43^	Iran	Cross-sectional	49.3±4.6	1538	Lipids	LDL-C and total cholesterol levels were higher in post- than in premenopausal women
Wang (2022)^25^	China	Longitudinal	Baseline: 50.9±10.4	281,319	DM	Postmenopausal women had higher risk of developing diabetes
Zhou (2019)^26^	China	Cross-sectional	49.4±8.1	569	10-year risk of CVD in DM	Menopause was associated with 10-year risk of CVD
Yu (2021)^20^	China	Cross-sectional	40–70	1352	MetS	Menopause was associated with higher prevalence of MetS
Feng (2008)^22^	China	Cross-sectional	44.8±7.4	9097	BMI, WHR, glucose, insulin, lipids, BP	Only WHR, TGs, total cholesterol, HDL-C and LDL-C were higher in post- than in premenopausal women
Wu (1990)^23^	China	Cross-sectional	40–54	598	BP, TC, TGs, HDL-C	Postmenopausal women had higher BP and lipid levels
Li (2019)^24^	China	Cross-sectional	40–70	3227	BMI, WC, BP, glucose, lipids, TP	Waist circumference, systolic and diastolic blood pressure, triglycerides, ALT, TP, and BUN were risk factors for DM in postmenopausal women.
Strand (2014)^19^	China	Cross-sectional	40–60	440	MetS	Prevalence of MetS was similar in pre- and postmenopausal women.
Blümel (2001)^57^	Chile	Longitudinal	Baseline: 40–60	271	BMI	BMI independent of menopausal differences.
Theodoro (2012)^29^	Brazil	Cross-sectional	40–65	617	WC, BMI	Postmenopause was associated with increased general obesity but not abdominal obesity when compared to premenopausal women.
Akl (2017)^31^	Brazil	Cross-sectional	47.7±5.8	273	MetS	No association between menopausal status and metabolic syndrome.
Fonseca (2019)^33^	Brazil	Cross-sectional	49.6±8.5	1916	Lipoprotein subfractions	Menopause was associated with TRL-C levels. Duration since menopause <2 years had the highest association with higher TRL-C and VLDL3-C.
Mendes (2013)^34^	Brazil	Cross-sectional	51.1±6.5	551	MetS	Menopause was associated with high blood pressure and elevated glucose levels.
Ghosh (2010)^58^	India	Cross-sectional	25–65	245	BMI, WC, WHR, total fat mass, fat free mass	Increased total fat mass, free fat mass, WC, and WHR in post- than premenopausal women.
Ghosh (2008)^45^	India	Cross-sectional	30–65	200	MetS	MetS was higher in postmenopausal women.
Dasgupta (2012)^44^	India	Cross-sectional	30–75	316	Lipids, glucose, BP	Postmenopausal stage was associated with elevated glucose, total cholesterol, triglycerides, LDL-C, and BP.
Dasgupta (2020)^46^	India	Cross-sectional	40–55	1400	BMI, BP	Menopause was associated with higher BMI and BP.
Sanchez-Rodriguez (2012)^52^	Mexico	Cross-sectional	40–60	374	Oxidative stress	Menopause was associated with oxidative stress as measured by the high lipoperoxide biomarkers.
Muchanga (2014)^53^	DRC	Cross-sectional	40–60	200	BP	Menopause was associated with prehypertension.
Setroame (2020)^54^	Ghana	Cross-sectional	47.7±16.8	185	MetS	Higher prevalence of metabolic syndrome in post- vs premenopausal women.
Jaff (2015)^55^	South Africa	Cross-sectional	40–60	702	BMI, WC, visceral fat, subcutaneous fat	No differences in BMI, WC, visceral, and subcutaneous fat between pre- and postmenopausal women.

a Articles used in the quantitative meta-analysis, Age expressed as mean ± standard deviation or range, AMH-anti-mullerian hormone, BP-blood pressure, BMI-body mass index, cIMT-carotid intima media thickness, CVD-cardiovascular disease, DM-diabetes mellitus, HDL-C-high density lipoprotein-cholesterol, HOMA-homeostatic model assessment for insulin resistance, LDL-C-low density lipoprotein-cholesterol, MetS-metabolic syndrome,,TGs-triglycerides, TP-total protein, TRL-C- triglyceride-rich lipoprotein-cholesterol, VLDL3-C- very-low-density lipoprotein cholesterol subfraction 3, WC-waist circumference, WHR-waist-to-hip ratio.

## Key Points

### Question/Objective

What is the association between menopause and cardiometabolic disease (CMD) risk factors in low- and middle-income countries (LMICs)?

### Findings

Forty-four articles were included in the systematic review and sixteen in the meta-analysis. The results showed that compared to premenopausal women, postmenopausal women had higher levels of metabolic syndrome, waist-to-hip ratio, blood pressure, triglycerides, and blood glucose but not general obesity, high-density lipoprotein cholesterol and carotid intima media thickness.

### Meaning

Menopause is associated with poor cardiometabolic health in LMICs; therefore, it is essential to increase public health awareness for monitoring and intervention of CMD risk factors in midlife women in these countries.
